# Residue propensities, discrimination and binding site prediction of adenine and guanine phosphates

**DOI:** 10.1186/1471-2091-12-20

**Published:** 2011-05-13

**Authors:** Ahmad Firoz, Adeel Malik, Karl H Joplin, Zulfiqar Ahmad, Vivekanand Jha, Shandar Ahmad

**Affiliations:** 1 Biomedical Informatics Center, PGIMER, Chandigarh-160012, India; 2 Department of Biological Sciences, East Tennessee State University, Johnson-City, TN, USA; 3 Department of Biology, Alabama A&M University, AL, USA; 4 Department of Nephrology, PGIMER, Chandigarh-160012, India; 5 National Institute of Biomedical Innovation, Osaka, Japan

## Abstract

**Background:**

Adenine and guanine phosphates are involved in a number of biological processes such as cell signaling, metabolism and enzymatic cofactor functions. Binding sites in proteins for these ligands are often detected by looking for a previously known motif by alignment based search. This is likely to miss those where a similar binding site has not been previously characterized and when the binding sites do not follow the rule described by predefined motif. Also, it is intriguing how proteins select between adenine and guanine derivative with high specificity.

**Results:**

Residue preferences for AMP, GMP, ADP, GDP, ATP and GTP have been investigated in details with additional comparison with cyclic variants cAMP and cGMP. We also attempt to predict residues interacting with these nucleotides using information derived from local sequence and evolutionary profiles. Results indicate that subtle differences exist between single residue preferences for specific nucleotides and taking neighbor environment and evolutionary context into account, successful models of their binding site prediction can be developed.

**Conclusion:**

In this work, we explore how single amino acid propensities for these nucleotides play a role in the affinity and specificity of this set of nucleotides. This is expected to be helpful in identifying novel binding sites for adenine and guanine phosphates, especially when a known binding motif is not detectable.

## Background

**Figure 1:**
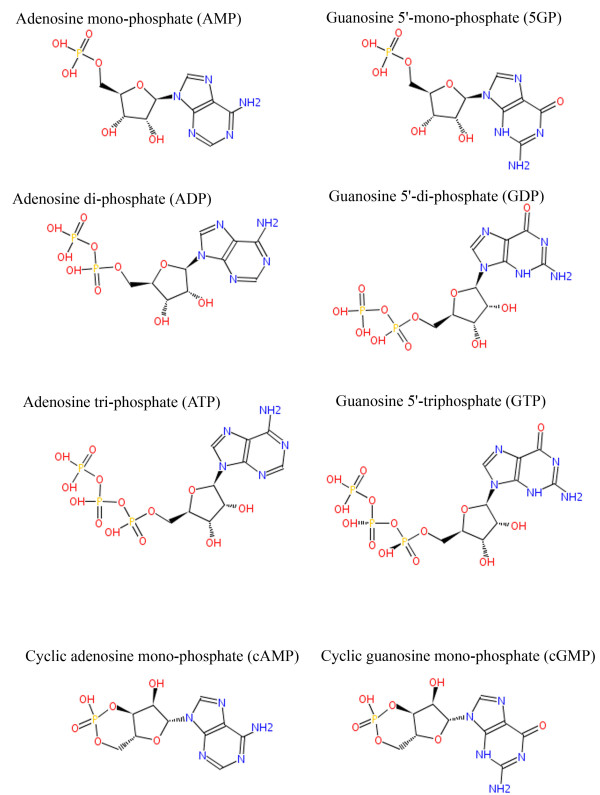
**Adenine and Guanine phosphates (nucleotides) found in complex with proteins in Protein Data Bank, with corresponding HETATM (three-letter) codes**.

Adenine triphosphate (ATP) is widely known to be energy currency of biological molecules as its conversion to corresponding di- and mono-phosphate leads to energy release, commonly used in conformational changes required for many biological interactions [1,2]. Closely related molecules such as guanidine triphosphate (GTP) also have similar metabolic implications [3,4]. Use of GTP versus ATP is highly specific to organisms as well as pathways [5]. Since, adenine and guanine have similar structures (both are purines) and essentially differ from each other by a nitrogenous versus oxygen group [5], (See Figure 1), a high degree of specificity between them is quite surprising and not well understood. A thorough understanding of this specificity therefore has wide biological implications, including discovery of metabolic drug targets as well as inhibitor design. There are other areas of biological research, where these molecules play a role such as cell-signaling and cofactor activity [6-11]. Thus, adenine and guanine phosphates form an important group of molecules, whose interactions with proteins at single residue as well as sequence and structural motifs levels have great significance but the process of this specificity lacks clear understanding. Discovery of binding sites for each of these molecules lies at the heart of this problem and it is essential to identify such binding sites for targeting inhibitors or understanding their function.

A typical problem of biochemical discrimination between ATP and ADP appears in the case of ATP synthesis, where the question is how does the enzyme bind hydrolyzed version of ATP (i.e. ADP plus orthophosphate HPO_5_, also called Pi), rather than ATP itself, into catalytic sites? In active cells, the cytoplasmic concentrations of ATP and Pi are approximately in the 2-5 mM range whereas that of ADP is at least 10-50-fold lower. Equilibrium binding assays have established that both ADP and ATP bind to catalytic sites of purified F1 and detergent solubilized F1Fo with relatively similar binding affinities (here F1 and Fo respectively refer to the catalytic and proton-translocating subunits of ATP synthase) [12,13]. Obviously, the enzyme must have evolved a specific mechanism for selectively binding ADP into catalytic sites while contemporaneously discouraging access of ATP during proton driven rotation and ATP synthesis. One hypothesis is that during ATP synthesis, proton gradient-driven rotation of subunits drives an empty catalytic site to bind Pi tightly, thus stereochemically precluding ATP binding and therefore selectively favoring ADP binding [14]. Thus, the fundamental unknown, "what is the molecular basis of the ability to bind ADP at catalytic sites of ATP synthase during ATP synthesis, in the face of a seemingly prohibitive [ATP/ADP] ratio," was solved by modifying the assay originally devised by Perez et al [15] in which the protection afforded by Pi against inhibition of ATPase activity, induced by covalent reaction of 7-chloro-4-nitrobenzo-2-oxa-1, 3,-diazol (NBD-Cl) with βY297, provides the measure of Pi binding. Their original work used mitochondrial inner membrane preparations; the successful application of the modified version of assay to both purified F1 and plasma membrane vesicle preparations from E. coli, resulted in identification of five Pi binding residues namely βArg-246, αArg-376, βLys-155, βArg-182, and αSer-347, and three non Pi binding residues namely, βAsn-243, αPhe-291, and αGly-351 [13,16-22].

ATP-binding sites have been typically identified by locating motifs in sequence and amongst them P-loop motif has been by far the most widely studied one [23-26]. Such motifs can be identified by sequence comparison; although the exact spacing between glycine residues implicated in these motifs is not always constant which may cause problems in identifying these motifs in novel proteins. However, the P-loop is not the only motif associated with ATP-binding so a motif based approach will not always work. It will obviously fail in cases where a binding site is not related to conserved motifs. Moreover the mere discovery of a motif does not help in understanding residue-wise interactions of proteins with ATP or its guanine analogues. Contribution of individual residues to the affinity of interactions cannot be inferred from such analysis.

A number of computational methods have been developed to identify ligand-binding sites in proteins at the residue level, using statistical and machine learning approaches where protein sequence information is the primary input for a prediction model [27-30]. In particular, models have been developed for DNA-binding and carbohydrate binding sites [27-31]. In regards to nucleotides, Saito et al. [31] used empirical scores for predicting nucleotide binding proteins which could successfully predict ATP binding sites. Recently, Chauhan et al., employed SVM to predict the ATP binding residues in ATP binding proteins using amino acid sequence and their evolutionary profiles [32]. In this work, we have developed support vector machine (SVM) based regression models for predictive and comparative analysis of adenine and guanine nucleotide binding sites in proteins. The analysis starts with the amino acid propensities for adenine and guanine phosphates which are then used to identify, which residues discriminate these similarly looking ligands. Then, machine learning methods are used to predict these binding sites directly from sequence data. Finally, the model trained on binding sites of adenine nucleotides is used to predict binding sites on guanine nucleotides and then to use these prediction strategies to discriminate between adenine and guanine recognition. Results of this study are likely to be helpful in annotating new proteins, their functional regions and select mutagenesis targets for a variety of molecular interactions.

## Methods

### Data sets

Figure 1 gives the overall structure and list of nucleotides considered in this study. As seen in this Figure, there are 4 pairs of adenine and guanine phosphates included here, corresponding to mono, di-, tri- and cyclic mono- phosphate versions of these bases. Numbers of overall and unique entries in Protein Data Bank (PDB) are listed in Table 1. In case of structures with multiple models (NMR-solved structures), only the first models were used and structures having a resolution lower than 2.5Å were discarded from the list of overall PDB entries. Additionally, structures having fewer than 30 residues were also removed from the dataset. Finally, all unique entries were obtained by removing redundancy at 30% sequence identity cutoff, so that in the final list no two proteins binding to the same ligand have more than 30% sequence similarity. Complete lists are provided in additional file Additional file 1.

**Table 1:** Adenine and Guanine phosphates in Protein Data Bank, considered in this study

Full name	HET name	PDB entries	Unique PDB entries	Number of BS	Number of NBS
Cyclic adenosine mono-phosphate	CMP	37	14	260	3757

Cyclic guanosine mono-phosphate	PCG	6	5	72	1041

Adenosine mono-phosphate	AMP	210	81	1274	25375

Adenosine di-phosphate	ADP	645	175	3144	59341

Adenosine tri-phosphate	ATP	369	131	2244	39468

Guanosine 5'-mono-phosphate	5GP	43	20	278	4749

Guanosine 5'-di-phosphate	GDP	333	64	912	19887

Guanosine 5'-triphosphate	GTP	121	33	606	10125

Here BS represents Binding sites (residues) where as NBS represents Non-binding sites (residues) respectively.

### Binding site

Residues in the selected proteins are labeled as binding and non-binding if any atom from that residue comes within 4.5Å distance with any atom of the nucleotide, when overall propensity values are considered. Atoms are grouped into main chain, side chain etc, for more detailed propensity calculations.

### Propensity

Propensity, P(i) for each of the 20 amino acids is calculated by pooling all the data from the selected proteins within a category (e.g. all ATP-binding proteins) and then taking the ratio of relative number of binding residues of that type ((*N_b_*(*i*)/*N*(*i*))) with the overall relative number of binding residues ((*N_b_*(*all*)/*N*(*all*))) i.e.

### Calculation of error bars

Multiple pseudo-copies of the entire data sets (for example all ATP-binding proteins) are created by successively and randomly picking up proteins one-by-one after replacement from the entire list (for example all ATP-binding proteins) until the total number becomes equal to the original data set. In this way, some proteins appear more than once whereas others are not selected at all. For each pseudo-copy of the data, propensity scores are computed and the standard deviation of these values is used as the error bar for each of the 20 amino acids. For the current study 500 copies of data were made for each category.

### Prediction method

All predictions are made using a five residue window composed of a row from position specific substitution matrix (PSSM) for each residue, resulting in a 21 × 5 = 105 dimensional input vector for each residue (20 dimensions for the identity of a residue and 21^st ^dimension for terminal position). Target sequences were scanned against the Non-redundant (NR) database of NCBI to compile a set of alignment profiles or position specific scoring matrices (PSSMs) using Position Specific Iterative BLAST (PSI BLAST) program [33]. Three cycles of PSI-BLAST were run for each protein and the scores were saved as profile matrices (PSSMs).

A sliding window is used to obtain predictions for all residues in a protein. The 105-dimensional vector inputs are trained using a Support Vector Regression model with default parameters as implemented in e1071 package of R programming environment (http://www.r-project.org). After trying a few runs with other kernels, we observed that Radial Basis Function (RBF) kernel performs the best. Thus all models used RBF kernel with default parameters. Target vectors consisted of one dimension, whose value corresponds to its binding state at a position (1 corresponding to binding, 0 otherwise). Entire data is trained using a Jackknife leave-one-protein-out procedure i.e. one protein is left out of the training set and SVM is trained for the remaining data. After the model is ready, the performance is tested on the left-out-protein. Finally reported values are the average over the proteins left out in each cycle, one after the other spanning an entire data set. This ensures that the reported performance represents true performance on blind data sets. However, when testing performance of models trained on data set of one ligand over the data sets of another ligand, performance of an SVM is also tested on the data on which it was trained (this includes all the data corresponding to that ligand). In most cases this score reaches 100% because SVM was able to over-learn and achieve a perfect separation on training examples. Apart from the SVM, we also tested the performance on neural network models trained using SNNS package [34]. However, SVM performance was found to be much better and no neural network results are discussed in this manuscript.

### Performance measure

A trained SVM regression model returns a real value between 0 and 1 which can be converted to a binary prediction of binding or non-binding at various cutoffs for each residue position. Predictions are called positive (P) if the output is more than a cutoff and negative (N) otherwise. If the positive and negative predictions correctly correspond to binding sites or correctly assign non-binding status to a residue, they are called True (T), otherwise False (F), thereby assigning to each residue (at a given cutoff) one of the four labels; viz., True positive (TP), False positive (FP), True negative (TN) and False negative (FN). The number of residues in each of the four categories is counted and the following scores are calculated:

Receiver operator characteristic (ROC) curve is plotted as a (1-specificity) versus sensitivity for all cutoffs and the area covered under this plot is known as area under the curve (AUC of ROC, or simply AUC) of prediction. For an ideal case, AUC reaches 1 or 100%, (this happens if for any cutoff, all residues correctly classified into binding and non-binding classes), whereas for a random case, the AUC values are about 0.5 (actual AUC can be lower than random, as prediction results correspond to a leave-one-out data, which is always different from the training data set). Thus, AUC gives an overall view of prediction performance and can be compared between various models. If the data is too unbalanced (few positive cases in a large data of negative cases), one may need to know the true positive out of a set of predictive positive cases (rather than true positive out of actually present positive cases as in the case of sensitivity). This is measured by precision:

A score called F-measure is often used to estimate predictive power of a model, which considers both sensitivity (also called recall) and precision. It is defined as, as the geometric mean of precision and recall i.e.

AUC and F-measure, along with the precision and recall at the best F-measure are included in the prediction results.

## Results and discussion

Four types of computations are performed in this work; (1) Residue propensities within adenine phosphates (2) Residue propensities within guanine phosphates (3) Comparison between adenine and guanine phosphate propensities and (4) Prediction performance for adenine and guanine phosphates and mutual similarity in prediction models. Results from these four analyses are presented and discussed in the following:

### Residue propensities within Adenine phosphates

**Figure 2:**
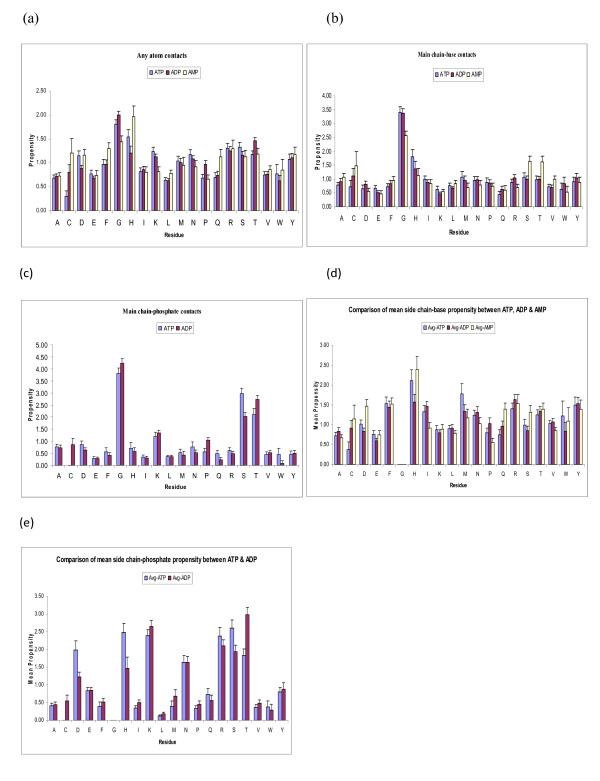
Amino acid propensities for various adenine phosphates (a) any contact between protein and nucleotide (b) protein main-chain contact with nucleotide base (c) protein main-chain contact with phosphate (d) protein side-chain contact with nucleotide base (e) protein side-chain contact with phosphate

Figure 2 shows residue propensities in mono-, di- and tri- nucleotides of adenine both in the overall (Figure 2(a)) as well as higher resolution contact definitions (Figure 2(b-e)). A number of observations can be made from these graphs.

1. Gly is the most abundant residue in all three cases. Role of Gly in forming ATP-binding P-loop is well known [35-37] and it is not surprising that this residue has the highest binding propensity to all three phosphates of adenine. It would be interesting to see, if there are any differences between the propensities of residues for the three phosphates.

2. In general residue propensities in three phosphates of adenine are very similar and hence the same binding sites are likely to recognize all three types, at least as far as single residue recognition goes. However, subtle differences do exist. Most prominent among them is the case of His residues, which have a higher propensity for AMP compared to ADP and ATP (Figure 2a). This is probably because His forms stacking interactions with adenine, which are facilitated by smaller phosphate tail (less hindrance). This hypothesis is supported by the fact that main chain contact propensities of His are quite low and hence the major contribution comes from its side chain. Further phosphate contact propensities of His side chain are also quite low, which is consistent with the above argument, as no stacking interaction is possible between His side chain and phosphate atoms. On the other hand Gly has a smaller propensity for AMP, probably because of the absence of a side chain, requiring longer tail in the nucleotide for interaction and hence forming fewer contacts with the nucleotide with the smallest phosphate tail. Again, in the absence of a side chain in Gly, all propensity comes from the main chain contacts and the overall preference of ATP contacts over AMP and ADP is retained (in comparison to main chain contacts of other residues).

### Residue propensities within Guanine phosphates

**Figure 3:**
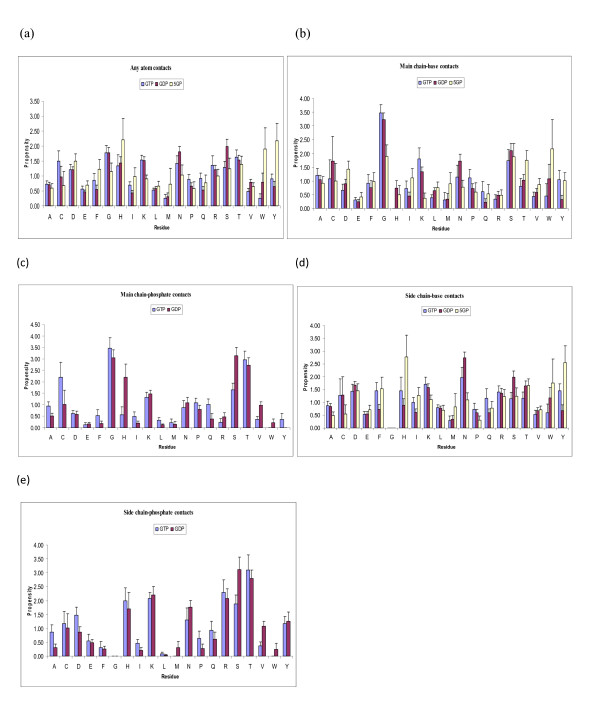
Amino acid propensities for various guanine phosphates (a) any contact between protein and nucleotide (b) protein main-chain contact with nucleotide base (c) protein main-chain contact with phosphate (d) protein side-chain contact with nucleotide base (e) protein side-chain contact with phosphate

Figure 3 shows residue propensities in mono-, di- and tri- nucleotides of guanine both in the overall (Figure 3(a)) as well as higher resolution contact definitions (Figure 3(b-e)). Main conclusions from these figures can be summarized as follows:

1. Residue propensities in guanine nucleotides have a distribution quite different from adenine phosphates (discussed in previous section). Most notable feature is that the difference between mono-, di- and triphosphates is much more prominent in guanine phosphates compared to adenine phosphates, as we see the propensity values for each residue type have different values for different phosphates.

2. At the atomic level His residues have been shown to have a preference for guanine, which is also confirmed by our analysis [38,39]. We also observe that His propensity for monophosphate is higher than di- and triphosphates, which is similar to the pattern in Adenine phosphates and could be due to the same reasons i.e. convenience of stacking interactions between imidazole rings of His and Guanine [40].

3. Tyrosine and Tryptophan propensities are the highest for GMP, and quite low for GDP and GTP, which together with His propensity values suggests that interactions between ring structures of Tyr, His and Trp are primary contacts between these residues and GMP, which are seriously impaired by the presence of long phosphate chain and hence do not occur in the case of GDP and GTP. This is also supported by strong differences between GMP and others in the case of side-chain-base contacts.

### Comparison between Adenine and Guanine phosphate propensities

**Figure 4:**
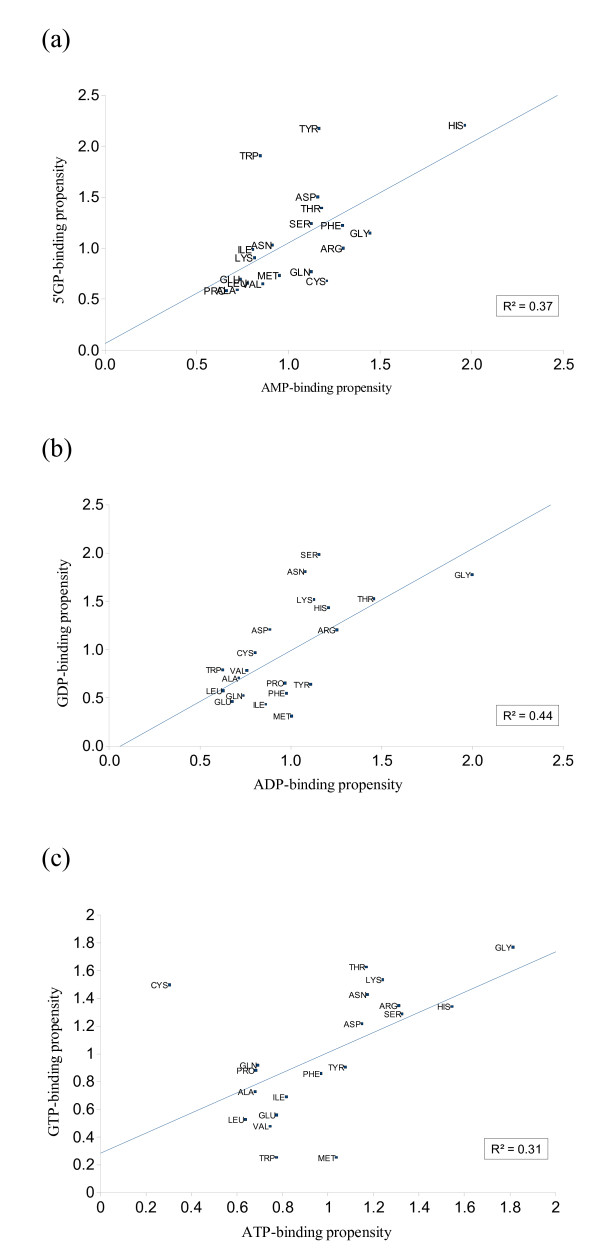
**Single residue propensity differences between similar adenine and guanine phosphates**. (a) mono-phosphate (b) diphosphates (c) triphosphates

Figure 4(a-c) shows the comparison of propensities between AMP, ADP, and ATP and their corresponding guanine phosphates. We observe that the correlation coefficient between these pairs ranges from 0.6 to 0.7 (R^2 ^= 0.37, 0.44 and 0.31 respectively for mono-, di- and triphosphates), which means that the two nucleotide pairs have strong similarity between them. However, the specificity is provided by the subtle differences, which do exist at single residue level. In particular, monophosphates are best distinguished by just two residues, Tyrosine and Tryptophan, which have a high preference for GMP, not observed in AMP. However, in the case of di- and triphosphates, hydrophilic residues prefer guanine and hydrophobic ones prefer adenine, as can be seen by the presence of more hydrophobic residues below the regression line in ATP versus GTP and ADP versus GDP plots (Figure 4(b-c)).

### Cyclic phosphates of adenine and guanine

**Figure 5:**
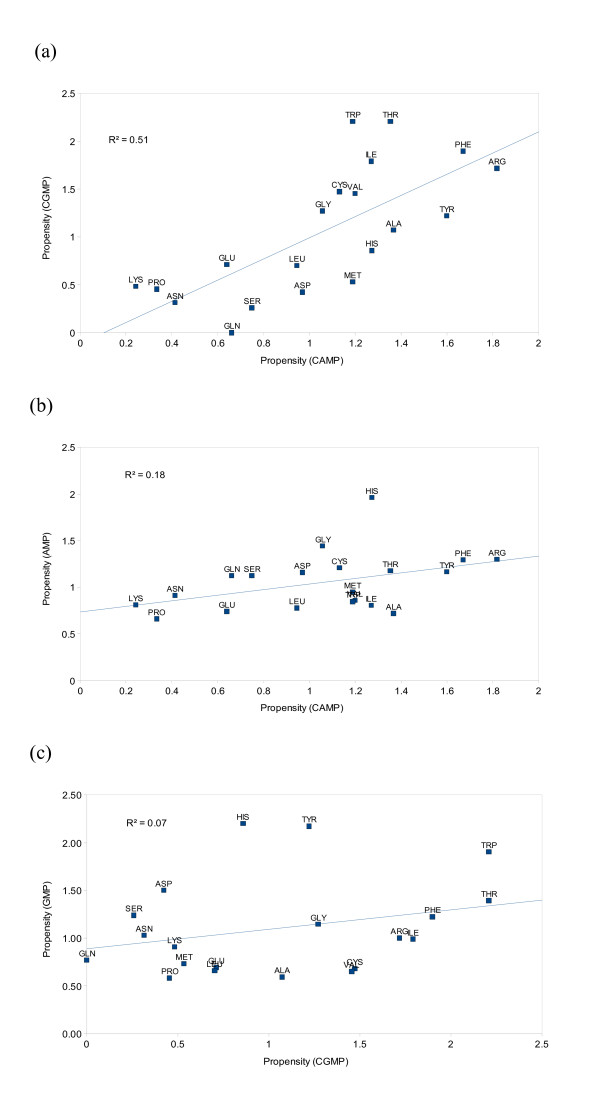
**Comparison of propensities between adenine and guanine phosphates with cyclic variants**. (a) cAMP versus cGMP (b) cAMP versus AMP (c) cGMP versus GMP

Figure 5(a) shows a comparison of propensities between cyclic monophosphates of adenine and guanine (cAMP and cGMP). Correlation coefficients between the propensities are also shown in Table 2. Despite a relatively small amount of data for cGMP, propensities values between the two cyclic ligands are very similar with a correlation coefficient (R = 0.72) higher than any pair of ligands in this study. In contrast the cyclic and noncyclic variants have relatively lower degree of correlation (R = 0.42 for adenine and 0.27 for guanine), as can be seen from parts (b) and (c) of Figure 5 also. Thus, it is quite clear that the cAMP and cGMP have close similarity in their residue-wise interactions, whereas despite having a similar nucleotide base, their non-cyclic versions are quite different. This highlights the crucial significance of the phosphate part of the ligand for interaction with ligands, as this part of the nucleotide distinguishes between the chemical natures of cyclic AMPs from non-cyclic ones.

**Table 2:** Comparison of propensity scores between cyclic and aliphatic adenine/guanine mono-phosphates

	Correlation (R)	**R**^**2**^
cAMP/cGMP	0.72	0.51
cAMP/AMP	0.42	0.18
cGMP/GMP	0.27	0.07

Within the cAMP and cGMP ligands, Trp residues continue to have a preference for guanine over adenine ligands, which is similar to what we observe in non-cyclic AMP and GMP (Figure 4a). However, His and Tyr residues, which have a higher GMP propensity than AMP, do not show that preference in cGMP over cAMP, highlighting a different mechanism of recognition in the case of these ligands. Interestingly, these two residues (His and Tyr) have a higher propensity for GMP than cGMP, which shows the significance of His and Tyr for interacting with GMP over any other guanine or adenine phosphate.

### Comparison with previously reported results

Although, the number of proteins as well as list of adenine and guanine derivatives considered in the present work is larger than the most significant relevant study, it would be interesting to draw comparison between the two works, wherever possible. However, it may be stated at the outset that the previous study was undertaken several years ago and did not contain all the proteins whose structures have become available since then, and hence included in the current study. Despite this expansion, many results reported earlier have been found to be robust enough to be confirmed by the current work. For example, aliphatic hydrophobic residues are either neutral or under-represented in binding sites, whereas aromatic residues overcome hydrophobicity-driven exclusion, probably due to their stacking preferences with aromatic rings of adenine and guanine. Previous study showed that Cys is preferred by guanine and not by adenine [5], which is confirmed by the current study. In addition, we show that smaller phosphate chain (mono-phosphate in contrast to triphosphate) allows a higher Cys propensity than its longer counterparts in adenine phosphates, as revealed by the order of propensity for Cys being AMP > ADP > ATP whereas the trend is the opposite for guanine, in which longer chain derivatives are preferred. Preferences and propensity trends of Arg, Trp & His residues are also consistent in the two studies. Similarly, overall preferences of charged and polar residues are also confirmed [5]. All results relating to cyclic variants of these ligands as well as comparison between mono-, di- and triphosphate are exclusive for this current study and not reported earlier.

### Prediction performance for adenine and guanine phosphates and mutual similarity in prediction models

A number of patterns are observed in the propensity data, which play a role in recognition of these ligands from other molecules as well as from one another. These propensity values are at a single residue scale and it is obvious that they are further constrained by structural and sequence neighbors in the actual binding sites. In this work, we focus on sequence-based predictions and therefore try to learn about the role of sequence neighbors in these interactions. To determine the extent to which neighbors influence interactions, we try to predict binding sites from sequence information for each ligand and monitor prediction performance. Subsequently, we try to see how far models trained on binding sites of one ligand can also predict binding sites of the other ligand. Ability of such trained models to make cross-predictions, would be a signal for their similarity and can be used to estimate the specificity of a pair of ligands as well as predict with some confidence binding sites of ligands for which sufficient training data was not available (e.g. cGMP).

Prediction performances of SVM-based models are shown in Table 3. Results indicate that all adenine phosphate binding sites can be predicted with AUC in the range of 80~85%. However, the performance for guanine phosphates is slightly poorer with AUC score being 74~83%. The lower prediction performance in guanine ligands shows that the binding sites in this class of ligands are less conserved than adenine phosphates and have a greater variety of interactions, which is not entirely determined by sequence environment. This greater flexibility may be helpful in nature's selection of guanine ligands as energy currency in some organisms over the other, whereas higher organisms go for a more robust recognition and probably exchange more energy. The best known example of this is enzyme ATP synthase, the fundamental means of cell energy production in animals, plants and almost all microorganisms. This enzyme is responsible for ATP synthesis by oxidative or photophosphorylation in membranes of bacteria, mitochondria, and chloroplasts. A typical 70 kg human with relatively sedentary lifestyle will generate around 2.0 million kg of ATP from ADP and Pi in a 75-year lifespan [21,41]. Further lowest performance is observed for cyclic version PCG, presumably because there is not enough data to train this model. This performance level is comparable to earlier reports on some of these systems, although our data sets and prediction strategy are different [32]. For example, we removed redundancy at 30% sequence identity compared to 40% used earlier. We also used a leave-one-out cross-validation instead of five-fold reported earlier. Both these strategies make the study more rigorous. More importantly, the number of ligands considered here is much more exhaustive and our study takes a comparative and analytic approach instead of a purely predictive perspective taken earlier. Nonetheless, the performance levels being very similar on the ligands which were studies earlier, some aspects of the current work may be considered a confirmation of previously published results.

**Table 3:** Overall prediction performance, measured by area under the curve (AUC) of ROC plots for binding sites of various ligands considered in this study

Ligand	Accuracy (%)	Sensitivity (%)	Specificity (%)	F-measure (%)	AUC (%)
					

**AMP**	86.7	62.3	87.5	39.6	80.1

**ADP**	91.6	63.4	93.2	60.2	84.7

**ATP**	90.2	63.4	91.8	52.8	82.7

**GMP**	78.4	77.7	79.0	45.7	75.8

**GDP**	87.0	75.0	87.9	59.2	82.4

**GTP**	84.8	62.5	86.3	40.9	74.5

**CAMP**	92.3	71.7	94.0	66.7	83.2

**PCG**	82.1	57.4	83.9	36.6	64.9

To estimate the similarity between the binding sites of adenine and guanine phosphates, a confusion matrix was constructed in which models trained on binding sites of one ligand were evaluated over the binding sites of the other. Table 4, shows an all-against-all comparison of ligands in this way. (For additional performance measures, see additional file Additional file 2). As expected the diagonal values in this matrix are all 100%, showing that the self-consistency-based model can over-learn from itself (Table 3 results are free from this bias, as they use cross-validation). All off-diagonal elements are significantly lower than 100%, as the trained and tested data sets belong to different ligands, yet their good prediction performance despite this difference is also quite visible. This result has two implications. First of all, it implies that all adenine and guanine phosphates have some common evolutionary rules (contained in the PSSM data used for predictions here), which separate binding sites from non-binding regions of protein sequences. Since, adenine and guanine have very similar structures [5] and their phosphate tails are also not drastically different, some common recognition elements are not totally unexpected after all. This similarity in models has an advantage when we want to know binding sites of any of these ligands, but poses a problem when specificity of one ligand over the other is required. Whatever information of specificity comes from these prediction models, translates only modestly into our ability to distinguish between binding sites of various ligands considered here. This is true despite the difference between propensities of single residues, probably because Trp, Tyr and His, which were shown above to confer specificity, suggesting they are not always responsible for the specific behavior and their difference in propensity is not enough in distinguishing between binding sites at a very high specificity. However, observed differences in diagonal and off-diagonal values may be valuable when a comparison is being made at a high throughput such as genome scale. It can significantly reduce the candidate residues for mutagenesis experiments and functional studies.

**Table 4:** Cross-prediction prediction AUC (in%) of trained models, measured by using model trained for binding sites of one ligand to predict binding sites of the other ligands

Tested	AMP	ADP	ATP	GMP	GDP	GTP	CAMP	PCG
**Trained**								
**AMP**	100.00	73.27	74.89	72.83	75.11	76.30	75.37	62.88
**ADP**	81.12	100.00	86.22	72.81	80.34	77.64	73.64	74.48
**ATP**	85.34	86.38	100.00	73.94	78.16	78.63	73.23	74.70
**GMP**	73.13	70.02	71.14	100.00	74.21	71.24	75.00	61.32
**GDP**	75.57	75.58	73.94	85.16	100.00	80.93	70.28	70.56
**GTP**	74.45	74.29	74.09	67.32	83.31	100.00	66.69	59.67
**CAMP**	70.17	69.77	70.15	66.96	69.19	70.92	100.00	91.20
**PCG**	64.86	65.00	64.64	62.56	66.09	65.61	77.82	100.00

Please note that all data of a given category was trained, allowing to over-learn on itself and hence self-prediction values (trained and test data being the same) for all models showed 100% correct predictions. This is in contrast to cross-validation performance results in Table 3.

## Conclusion

Adenine and guanine phosphates recognize binding sites on proteins at a single residue level as well as complex sequence neighbor effects. Using evolutionary profiles of proteins, binding sites corresponding to these ligands can be predicted with good confidence, yet the predictability of binding sites of one of them in contrast to other remains a challenging problem. Although, encouraging results are obtained using current approach, more work is needed to improve performance of predicting specificity of various adenine and guanine phosphates with respect to one another.

## Authors' contributions

SA, ZA, and KJ planned the project and SA coordinated it. The study was implemented by AM and AF with advice from VJ. All SVM were trained and analyzed by SA. AM and SA prepared the manuscript, with AM in the lead and each author advised on it. All authors read and approved the final manuscript.

## Supplementary Material

Additional file 1 **List of unique PDB ids used in the current study**. The file contains the list of adenine and guanine binding proteins in Protein Data Bank that were considered in this study. Click here for file

Additional file 2 **Prediction performance of SVM models trained on a data set of one ligand and tested on the other**. All data of a given category was trained, allowing over-learning on it and hence self-prediction values (trained and test data being the same), all models showed 100% correct predictions. Click here for file
