# Supplementary material for: Residue propensities, discrimination and binding site prediction of adenine and guanine phosphates
Source: BMC Biochem. 2011 May 13;12:20. doi: 10.1186/1471-2091-12-20 (PMC3113737; doi:10.1186/1471-2091-12-20)
Supplement: Additional file 1 — List of unique PDB ids used in the current study. The file contains the list of adenine and guanine binding proteins in Protein Data Bank that were considered in this study. [file 1471-2091-12-20-S1.PDF]

Table S1
List of unique PDB ids used in the current study:

ADP	ATP	AMP	CMP	GDP	GTP	5GP	PCG
13PKA	1A49A	2F3DA	1G6NA	1A2KC	1A8RA	1QK3A	1BE4A
1A9XA	1A82A	1HTOA	1Q43A	1DARA	1C80A	2BLEA	1MC0A
1B4SA	1ATPE	1TB5A	1XFWA	1G4US	1CKMA	2CNWA	1Q3EA
1B62A	1AYLA	2PZAA	2OURA	1G7SA	1FRWA	1VCZA	1RI3A
1B6SA	1B0UA	2J91A	1Z5UA	1GRNB	1GWNA	2QORA	2GX5C
1BS1A	1B8AA	1O94C	1SK6A	1H2TZ	1GX5A	1C9KA	
1CZAN	1CSNA	1UKZA	1YKDA	1H65A	1HWXA	1SGXA	
1DJNA	1DV2A	2GM3A	2PW3A	1HE1A	1JLRA	1T9SA	
1E19A	1DY3A	2R7MA	1CX4A	1JNYA	1KSGA	2B8WA	
1E1RD	1E2QA	1OREA	2ZMFA	1JWYB	1LOOA	1PZMA	
1E9FA	1E79A	1CT9A	1NHKL	1MKYA	1NRJB	2FXVA	
1EHIA	1E8XA	1GPMA	1VP6A	1MREH	1RA7A	1HGXA	
1EQMA	1EE1A	1MB9A	1LB2B	1MREL	1T91A	1G7CA	
1F48A	1EQYA	1ECJA	1LPCA	1N7HA	1UVKA	1JXMA	
1FNNA	1ESQA	2QGAB		1O87A	1W5AA	2PNZB	
1FP6A	1F2UA	1J20A		1OFUA	1WA5C	2PNZA	
1FWKA	1F2UB	2GSUA		1OW3A	1WDTA	1ZNXA	
1G3QA	1F9AA	1YXUA		1R8SE	1YR8A	2YWTA	
1G41A	1FMWA	1FA9A		1S4OA	2A5DA	1RNCA	
1G6HA	1G5TA	1C0AA		1SIWA	2A8SA	3RHNA	
1G6OA	1GN8A	1V26A		1SVIA	2BTOA		
1GC5A	1GTRA	1UXNA		1T2AA	2BTOT		
1GKZA	1GZ3A	1K9YA		1TPZA	2CLSA		
1GSAA	1HP1A	2VARA		1VJ7A	2FB3A		
1HI5A	1I7LA	1OBDA		1VJJA	2FH5B		
1HTWA	1II0A	2A7XA		1WQ1G	2FP4B		
1HW8A	1J09A	2ZE5A		1XJEA	2H57A		
1I58B	1J1ZA	2GXQA		1YRAA	2IRXA		
1IAHA	1J7KA	2UV4A		1ZCBA	2OH5A		
1IN4A	1JJVA	2DCLA		1ZNYA	2PXAA		
1IOVA	1KJ8A	2FJBA		2AN9A	2Q0EA		
1J1CA	1KO5A	1AMUA		2CVWA	2QEYA		
1J7LA	1KP2A	2D1QA		2CXXA	2QV6A		
1K3CA	1KP8A	3CJ7A		2DBYA			
1KK8A	1KVKA	2YRXA		2DXEA			
1LTQA	1M83A	2QJTA		2DYKA			
1LVGA	1MB9A	1Z84A		2E87A			
1MP8A	1MJHA	3C0HA		2EFCA			
1MWMA	1N48A	1Y1PA		2G77A			
1N06A	1N5IA	2OUNA		2GEKA			
1NY5A	1NGEA	12ASA		2GJ9A			
1O51A	1NSFA	2V8QE		2HCJA			
1O6BA	1OBDA	2HCRA		2HCJB			
1OFHA	1PK8A	2Q2TA		2HEKA			
1OH6A	1Q97A	2F17A		2I8UA			
1OH9A	1QHGA	1EFVB		2J1LA			
1OXUA	1QHHA	1QB8A		2NZXA			
1P72A	1QHHB	1TBWA		2OGIA			
1PFKA	1QHHD	2AK3A		2OM2B			

1Q8YA	1QHXA	1ANKA	2P8YT
1Q9SA	1R8BA	1KTGA	2PHNA
1R6BX	1S9JA	1Z6SA	2QF2A
1RDQE	1SU2A	2OWOA	2QM7A
1RZUA	1SVMA	1RY2A	2QN6A
1SQ5A	1TC0A	1ZJWA	2QU8A
1SVLA	1TIDA	2CFMA	2R6R1
1T3TA	1TIDD	1MF0A	2RCNA
1T6XA	1U5RA	1UA4A	2V40A
1TC6A	1U5VA	2I4IA	2VAPA
1TH8A	1VC9A	1ISOA	2YV5A
1TY8A	1VJCA	1HDIA	2YWHA
1TZDA	1W7AA	2C5SA	2ZEJA
1U0JA	1WKLB	2HBLA	2ZGYA
1UC9A	1X01A	2QRKA	3BH7B
1UKYA	1XDNA	2EQAA	
1UW1A	1XDPA	1JP4A	
1VA6A	1XEFA	1NH8A	
1VHLA	1XEXA	2ARTA	
1W44A	1XMIA	1JWBB	
1W5SA	1XNGA	1S68A	
1W78A	1Y8QA	3BERA	
1WBPA	1Y8QB	1VD1A	
1WNLA	1YFRA	1W0HA	
1WPGA	1YUNA	2YVOA	
1X3MA	1Z0SA	1V8SA	
1X6VB	1ZAOA	1UUYA	
1XJKA	1ZP9A	1RAOA	
1XMVA	2A84A	2G1UA	
1XRJA	2AQXA	1KPFA	
1XW4X	2ARUA	2GMKA	
1XX6A	2BEKA	1JWBD	
1Y63A	2BIYA		
1Y8OA	2BU2A		
1YP4A	2C01X		
1YQTA	2C8VA		
1Z2NX	2C96A		
1Z5AA	2CJAA		
1Z6TA	2E5YA		
1ZARA	2E89A		
1ZS6A	2F02A		
1ZTHA	2FAQA		
2A2CA	2FSGA		
2AKOA	2HMUA		
2AWNA	2HVYA		
2AXNA	2HVYB		
2B9FA	2I4OA		
2BEJA	2IAJA		
2BFRA	2IDXA		
2BVCA	2IJMA		
2C2AA	2IVPA		
2C31A	2IXEA		

2C98A	2IYWA
2C9OA	2J3MA
2CDNA	2J9CA
2CDUA	2J9LA
2CE7A	2NT8A
2CGJA	2O0HA
2CN5A	2OGXA
2CNQA	2OH5A
2CVXA	2P09A
2D0OA	2Q0DA
2D7DA	2Q66A
2DPYB	2Q7GA
2DR3A	2Q97T
2DWCA	2QB8A
2DY9A	2QK4A
2E2PA	2QRDE
2F1JA	2QUIA
2FNAA	2QXLA
2FSIA	2R7LA
2FV7A	2V7QE
2GK6A	2VHQA
2GKSA	2YWWA
2GL6A	2YXUA
2GR0A	2Z02A
2GRYA	2Z08A
2H1FA	2Z1UA
2HGSA	3BJUA
2HMVA	3BU5A
2HV7B	3C5EA
2HXHC	3C9RA
2IF8A	
2IO8A	
2IS6A	
2IW3A	
2IYQA	
2J0WA	
2J9DB	
2JCBA	
2JFGA	
2JGVB	
2KINA	
2NO0A	
2NUNA	
2O0JA	
2O1VA	
2OBMA	
2OJWA	
2OLJA	
2ONMA	
2OXCA	
2P9IA	
2PL3A	

2PO0A
2PYWA
2Q14A
2Q2RA
2QB5A
2QRDB
2QRDE
2QSYA
2QWLA
2QZ4A
2R7NA
2RIOA
2V1XA
2V2ZA
2V7YA
2YWVA
2YX6B
2Z0HA
3BRBA
3C4NA
3C4ZA
3C9UA
