# Supplementary material for: Residue propensities, discrimination and binding site prediction of adenine and guanine phosphates
Source: BMC Biochem. 2011 May 13;12:20. doi: 10.1186/1471-2091-12-20 (PMC3113737; doi:10.1186/1471-2091-12-20)
Supplement: Additional file 2 — Prediction performance of SVM models trained on a data set of one ligand and tested on the other. All data of a given category was trained, allowing over-learning on it and hence self-prediction values (trained and test data being the same), all models showed 100% correct predictions. [file 1471-2091-12-20-S2.PDF]

Table S2: Prediction performance of SVM models trained on a data set of one ligand and tested on the other. Please note that all data of a given category was trained, allowing to over-learn on itself and hence self-prediction values (trained and test data being the same), all models showed 100% correct predictions. This is in contrast to cross-validation performance results in Table 3 (in the manuscript).

Trained	Tested	Accuracy (%)	Sensitivity (%)	Specificity (%)	F-measure (%)	AUC (%)
**AMP**	AMP	100.0	100.0	100.0	100.0	100.0
**AMP**	ADP	91.5	28.7	94.9	25.5	73.3
**AMP**	ATP	89.0	41.3	91.7	28.7	74.9
**AMP**	GMP	93.2	23.6	97.0	26.3	72.8
**AMP**	GDP	83.6	54.7	84.9	22.7	75.1
**AMP**	GTP	86.1	47.7	88.4	27.7	76.3
**AMP**	CAMP	84.7	43.8	87.5	26.5	75.4
**AMP**	PCG	86.1	32.0	89.9	23.0	62.9
**ADP**	AMP	96.2	25.3	98.1	26.0	81.1
**ADP**	ADP	100.0	100.0	100.0	100.0	100.0
**ADP**	ATP	96.2	41.9	99.2	54.0	86.2
**ADP**	GMP	84.3	47.5	86.3	23.7	72.8
**ADP**	GDP	95.5	27.2	98.7	34.8	80.3
**ADP**	GTP	90.3	35.6	93.6	29.1	77.6
**ADP**	CAMP	84.3	40.4	87.2	24.4	73.6
**ADP**	PCG	88.3	46.0	91.3	33.8	74.5
**ATP**	AMP	96.5	31.3	98.3	32.6	85.3
**ATP**	ADP	96.0	45.2	98.7	53.1	86.4
**ATP**	ATP	100.0	100.0	100.0	99.9	100.0
**ATP**	GMP	84.0	52.5	85.7	25.2	73.9
**ATP**	GDP	94.7	20.0	98.2	25.1	78.2
**ATP**	GTP	90.5	39.1	93.6	31.5	78.6
**ATP**	CAMP	91.8	23.3	96.4	26.4	73.2
**ATP**	PCG	92.0	46.0	95.2	42.6	74.7
**GMP**	AMP	83.9	42.5	85.0	12.4	73.1
**GMP**	ADP	83.8	36.3	86.3	18.4	70.0
**GMP**	ATP	84.5	35.7	87.3	19.9	71.1
**GMP**	GMP	100.0	100.0	100.0	100.0	100.0
**GMP**	GDP	83.8	48.0	85.4	20.7	74.2

**GMP**	GTP	80.4	47.4	82.4	21.3	71.2
**GMP**	CAMP	77.4	55.4	78.9	23.6	75.0
**GMP**	PCG	83.8	38.0	87.0	23.3	61.3
**GDP**	AMP	82.3	49.5	83.2	13.0	75.6
**GDP**	ADP	92.0	26.6	95.5	25.0	75.6
**GDP**	ATP	86.1	38.3	88.9	22.9	73.9
**GDP**	GMP	94.6	29.3	98.1	35.9	85.2
**GDP**	GDP	100.0	100.0	100.0	99.8	100.0
**GDP**	GTP	95.2	26.7	99.3	38.4	80.9
**GDP**	CAMP	81.6	39.6	84.4	21.3	70.3
**GDP**	PCG	91.5	38.0	95.2	36.5	70.6
**GTP**	AMP	82.3	49.5	83.2	13.0	74.4
**GTP**	ADP	91.1	25.8	94.6	22.6	74.3
**GTP**	ATP	84.5	43.6	86.8	23.2	74.1
**GTP**	GMP	80.9	40.9	83.1	18.1	67.3
**GTP**	GDP	95.5	33.3	98.4	39.7	83.3
**GTP**	GTP	100.0	100.0	100.0	99.9	100.0
**GTP**	CAMP	67.2	57.9	67.8	18.2	66.7
**GTP**	PCG	81.6	36.0	84.8	20.2	59.7
**CAMP**	AMP	76.9	48.3	77.6	10.0	70.2
**CAMP**	ADP	78.2	44.8	80.0	17.2	69.8
**CAMP**	ATP	76.0	50.6	77.4	18.5	70.2
**CAMP**	GMP	74.6	52.9	75.8	17.6	67.0
**CAMP**	GDP	78.6	51.8	79.8	17.5	69.2
**CAMP**	GTP	77.7	56.1	79.0	21.9	70.9
**CAMP**	CAMP	100.0	100.0	100.0	100.0	100.0
**CAMP**	PCG	95.3	76.0	96.7	67.9	91.2
**PCG**	AMP	66.7	57.6	67.0	8.5	64.9
**PCG**	ADP	65.1	58.1	65.5	14.4	65.0
**PCG**	ATP	62.4	64.0	62.3	15.5	64.6
**PCG**	GMP	60.2	68.6	59.7	15.0	62.6
**PCG**	GDP	71.2	55.7	71.9	14.5	66.1
**PCG**	GTP	68.6	60.0	69.1	17.6	65.6
**PCG**	CAMP	92.5	30.0	96.6	33.3	77.8
**PCG**	PCG	100.0	100.0	100.0	100.0	100.0
